# Supplementary material for: Microbial metabolite sensor GPR43 controls severity of experimental GVHD
Source: Nat Commun. 2018 Sep 10;9:3674. doi: 10.1038/s41467-018-06048-w (PMC6131147; doi:10.1038/s41467-018-06048-w)
Supplement: Supplementary file 1 — Supplementary Information [file 41467_2018_6048_MOESM1_ESM.pdf]

Supplementary Information

Microbial metabolite sensor GPR43 controls severity of experimental GVHD

Fujiwara et al

Supplementary Figure1

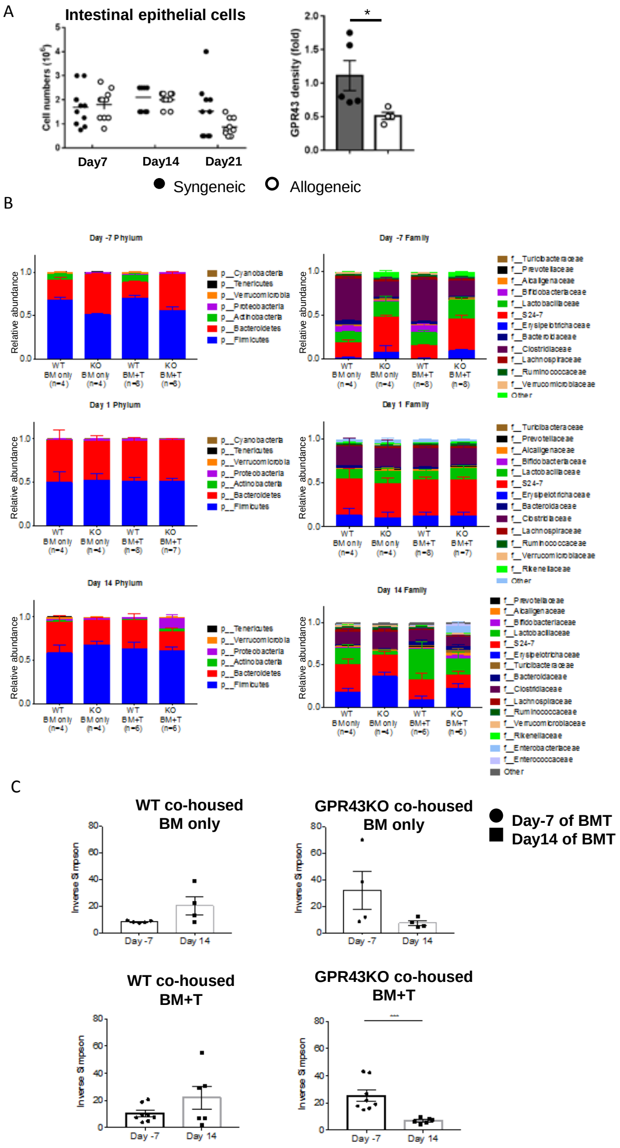

Supplementary Figure1: GPR43$^{-/-}$ mice increased abundance of Proteobacteria. **A**, B6 WT mice received BMT from either syngeneic B6 or allogeneic BALB/c donors. IEC numbers from syngeneic and allogeneic WT B6 recipients 7, 14 and 21 days after BMT (left, n=10 each, two-tailed unpaired *t*-test). Densitometric analysis of GPR43 normalized to IEC numbers from syngeneic and allogeneic WT B6 recipients 14 days after BMT (right, n=6 syngeneic, n=4 allogeneic, two-tailed Mann-Whitney *U* test). **B-C,** B6 WT and *Gpr43*$^{-/-}$ mice (co-housed for 7 days before BMT) received BMT from allogeneic 129S1 donors. Stool specimens obtained from WT and *Gpr43*$^{-/-}$ mice were collected on day -7, +1, and +14 after BMT, and analyzed by 16S rRNA gene sequencing. **B**, Comparison of bacterial species abundance determined by taxonomy. **C**, Diversity of microbial floras from co-housed was determined by the inversed Simpson index (n=4 BM only each, n=8 BM+T Day-7 each, n=6 BM+T Day 14, two-tailed Mann-Whitney *U* test). *$P < 0.05$, *** $P < 0.001$, error bars show the mean ± s.e.m.

Supplementary Figure2

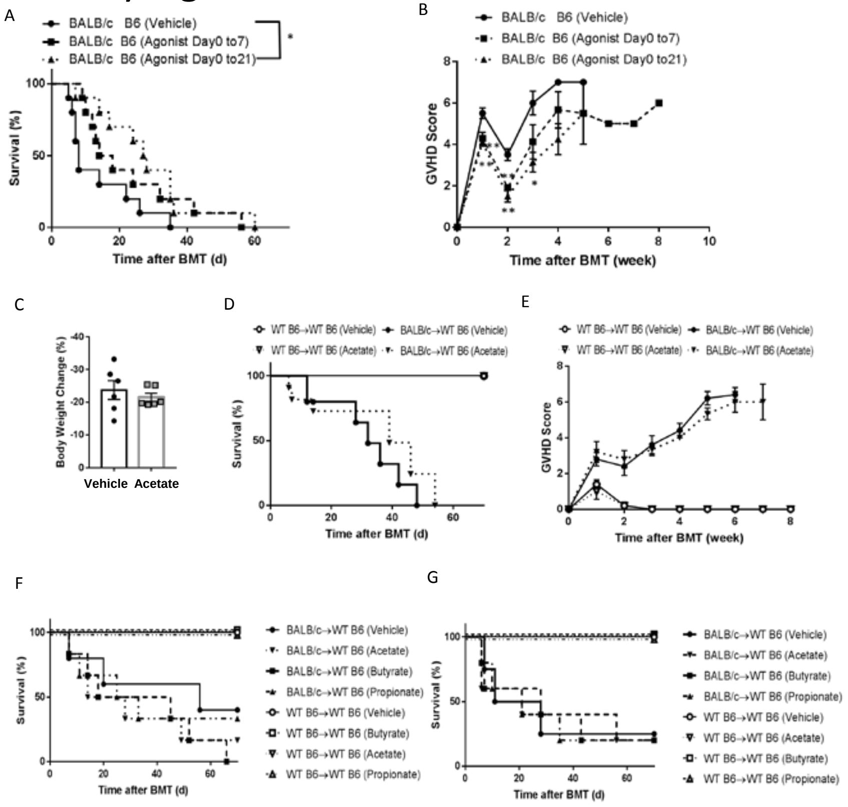

Supplementary Figure2: High amounts of short-chain fatty acids fail to ameliorate GVHD. A-B, WT B6 mice received BMT from allogeneic BALB/c donors treated with intragastric vehicle or GPR43 agonists (10mg kg^{-1} per day) for indicated periods (left, n=10 each, log-rank test for survival, two-tailed Mann-Whitney *U* test for GVHD Score). **C-G,** WT B6 mice received BMT from either syngeneic B6 or allogeneic BALB/c donors treated with intragastric vehicle, acetate, butyrate or propionate from day 0 to day 21. **C**, **D**, **E**, Body weight change on day 21, survival and clinical GVHD score after BMT for WT B6 recipients treated with intragastric vehicle or acetate (15mg kg^{-1} per day) from day 0 to day 21 are depicted (two-tailed unpaired *t*-test for body weight change, log-rank test for survival, two-tailed Mann-Whitney *U* test for GVHD Score). Data are representative of three experiments, each with n = 6 mice per group. **E**, Survival after BMT is shown for vehicle, acetate (150mg kg^{-1} per day), butyrate (100mg kg^{-1} per day) or propionate (150mg kg^{-1} per day) treatments (n=5 syngeneic each and allogeneic Vehicle, n=6 Butyrate, Acetate and Propionate each, log-rank test). **G**, Survival after BMT for vehicle, acetate (1500mg kg^{-1} per day), butyrate (1000mg kg^{-1} per day) or propionate (1500mg kg^{-1} per day) treatments is shown (n =5 mice per group (log-rank test for survival). Data are representative of three experiments. *$P < 0.05$, ** $P < 0.01$, error bars show the mean ± s.e.m.

Supplementary Figure3

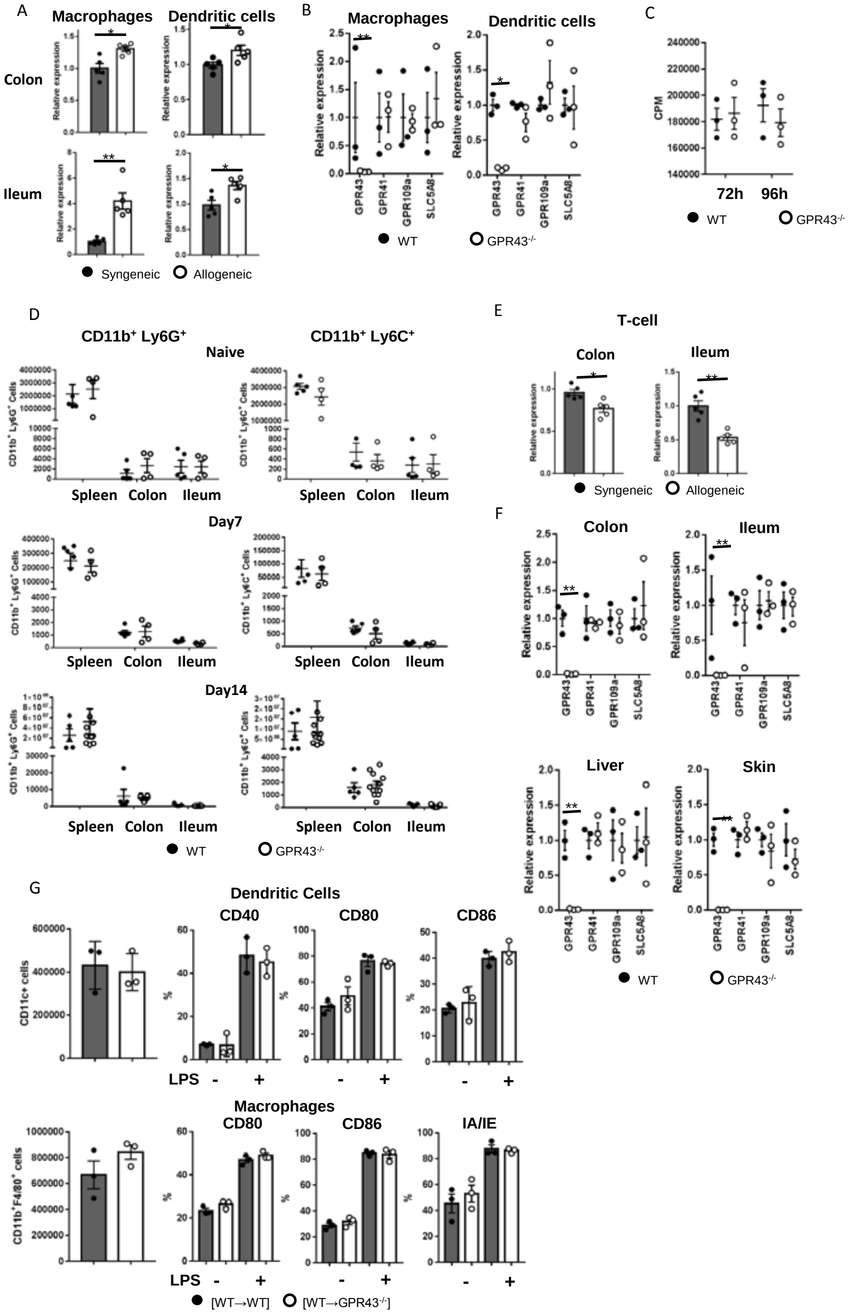

Supplementary Figure 3 Cont

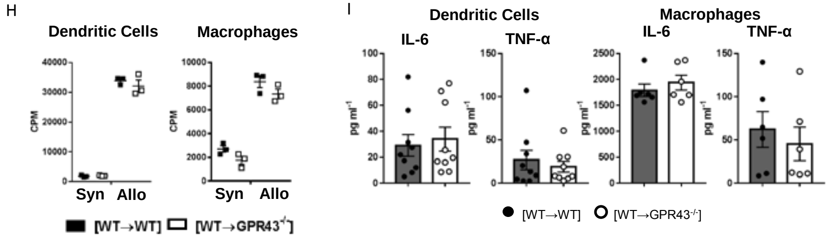

Supplementary Figure3: GPR43 deficiency does not cause compensatory changes in SCFA receptor expression nor inhibition of allogeneic T cell proliferation. A. WT B6 mice received BMT from either syngeneic B6 or allogeneic BALB/c donors. Gene expression of *Gpr43* in isolated macrophages and dendritic cells from colon and ileum on day 7 after BMT (n=5, two-tailed Mann-Whitney *U* test). **B**, Gene expression of *Gpr43, Gpr41, Gpr109a* and *Slc5a8* in peritoneal macrophages and splenic DCs from WT B6 and $Gpr43^{-/-}$ mice (n=3, two-tailed Mann-Whitney *U* test). **C**, Isolated T cells from BALB/c were cultured with irradiated splenic DCs from WT B6 and $Gpr43^{-/-}$ animals for 72 and 96 hours in an MLR and analyzed for proliferation following ^{3}H-thymidine incorporation during the last 16 hours of incubation (n=3). **D**, Total number of neutrophils ($CD11b^+$ $Ly6G^+$) and monocytes ($CD11b^+$ $Ly6C^+$) in spleen, colon and ileum from WT B6 or $Gpr43^{-/-}$ recipients 0, 7 and 14 days after allogeneic BMT (n=5 WT, n=4 $Gpr43^{-/-}$). **E**, Gene expression of *Gpr43* in isolated T cells from colon and ileum on day 7 after BMT (n=5, two-tailed Mann-Whitney *U* test). **F**, Gene expression of *Gpr43, Gpr41, Gpr109a* and *Slc5a8* in colon, ileum, liver and skin from WT B6 and $Gpr43^{-/-}$ mice (n=3, two-tailed Mann-Whitney *U* test). **G-I**, Chimeric [WT B6→WT B6] and [WT B6→ $Gpr43^{-/-}$] animals received 900 cGy. Dendritic cells ($CD11c^+$) from spleen and macrophages ($CD11b^+$ $F4/80^+$) from peritoneal cavity were isolated 72hrs after irradiation. **G**, Total number of dendritic cells and macrophages. CD40, CD80, CD86 positive populations in dendritic cells and CD80, CD86, IA/IE positive populations in macrophages with LPS treatment (n=3 each). **H**, Isolated T cells from WT B6 or BALB/c were cultured with isolated dendritic cells in spleen and isolated macrophages in peritoneal for 72h in an MLR and analyzed for proliferation following ^{3}H-thymidine incorporation during the last 6 hours of incubation (n=3). **I**, TNFα and IL-6 production by dendritic cells and macrophages stimulated for 6h with LPS as measured by ELISA (n=9 DCs, n=6 Macrophages, pooled from 2-3 experiments). *$P < 0.05$, ** $P < 0.01$, error bars show the mean ± s.e.m.

Supplementary Figure 4

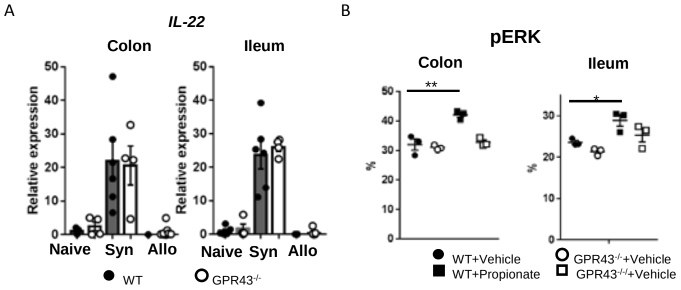

Supplementary Figure4: Extracellular signal-regulated Kinase (ERK) plays a crucial role in inflammasome activation following GPR43 stimulation. A, B6 WT and *Gpr43*$^{-/-}$ mice received BMT from either syngeneic B6 or allogeneic BALB/c donors. Gene expression of *IL-22* in intestine lamina propria cells of colon and ileum from naïve, syngeneic and allogeneic recipients at day14 after BMT (n =5 WT naïve and allogeneic, n=4 *Gpr43*$^{-/-}$ naïve and syngeneic, n=10 *Gpr43*$^{-/-}$ allogeneic). **B**, WT B6 and *Gpr43*$^{-/-}$ mice received oral administrations of vehicle or propionate (15mg kg^{-1} per day) for 3 days. Percentage of phosphorylated ERK positive IECs after treatment (n=3, each, two-tailed unpaired *t*-test) is shown. Data are representative of two experiments. *$P < 0.05$, ** $P < 0.01$, error bars show the mean ± s.e.m.

Supplementary Figure5

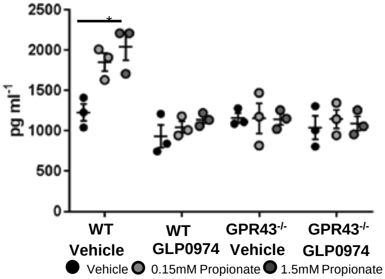

Supplementary Figure5: GPR43 antagonist inhibited GPR43 signals in IECs. IL-18 production by colon and ileum explant culture from WT B6 and *Gpr43*$^{-/-}$ mice stimulated overnight with GPR43 antagonist (GLPG0974, 1uM), propionate or butyrate (indicated concentration) as measured by ELISA (n=3 each, two-tailed unpaired *t*-test). Data are representative of two experiments. * $P < 0.05$, error bars represent the mean ± s.e.m.

Supplementary Figure6

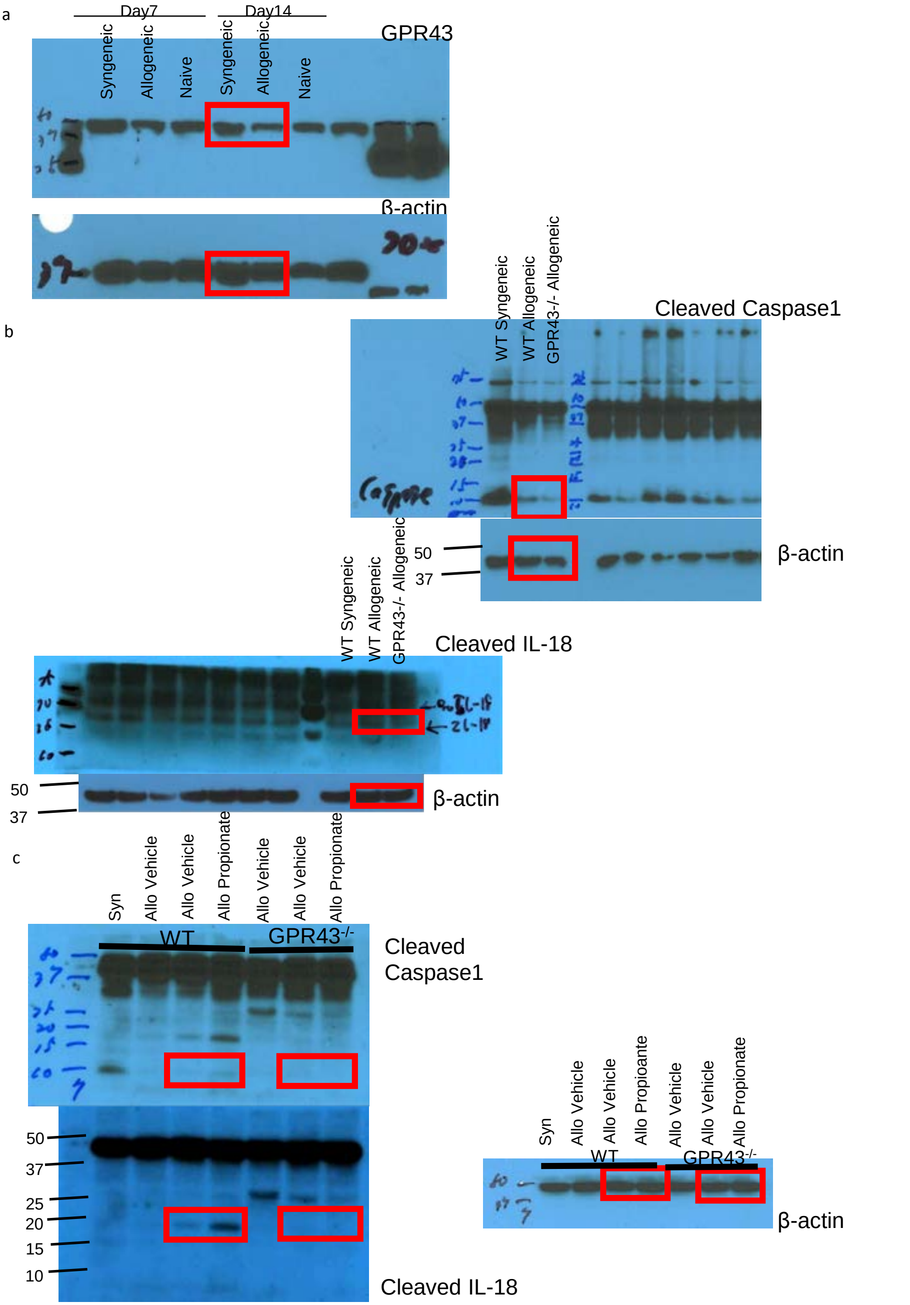

Supplementary Figure6: Uncropped immunoblotting images from Figure 1B (a), 5A (b) and 5C (c). The red boxes indicate the specific bands presented in each Figure.

Supplementary Figure7

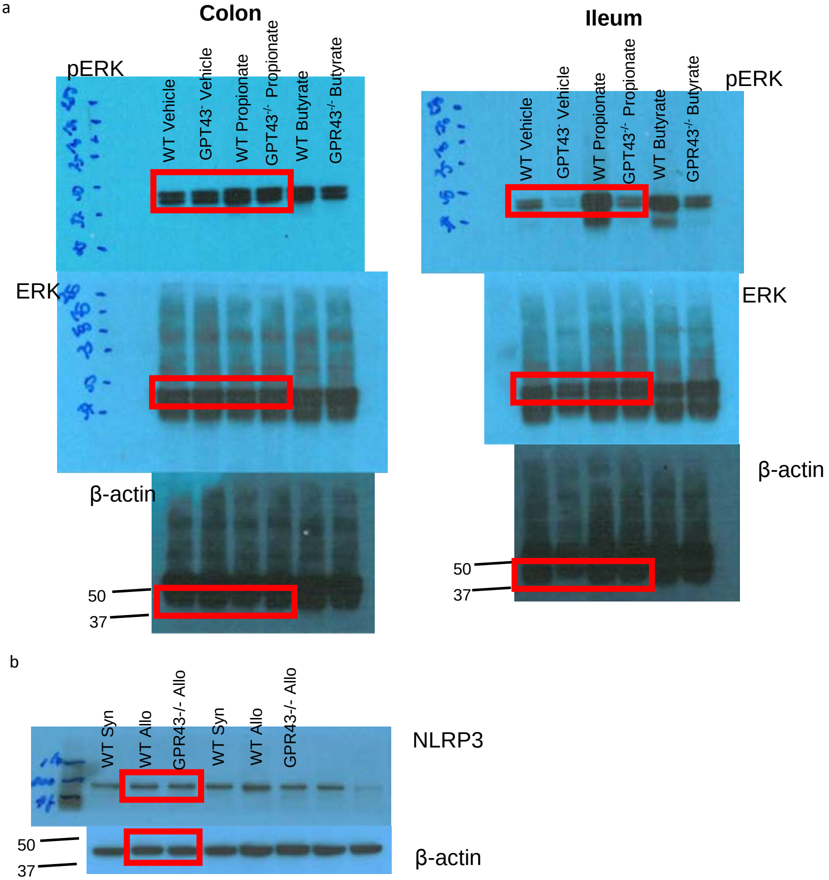

Supplementary Figure7: Uncropped immunoblotting images from Figure 5G (a) and 6A (b). The red boxes indicate the specific bands presented in each Figure.

Supplementary Figure8

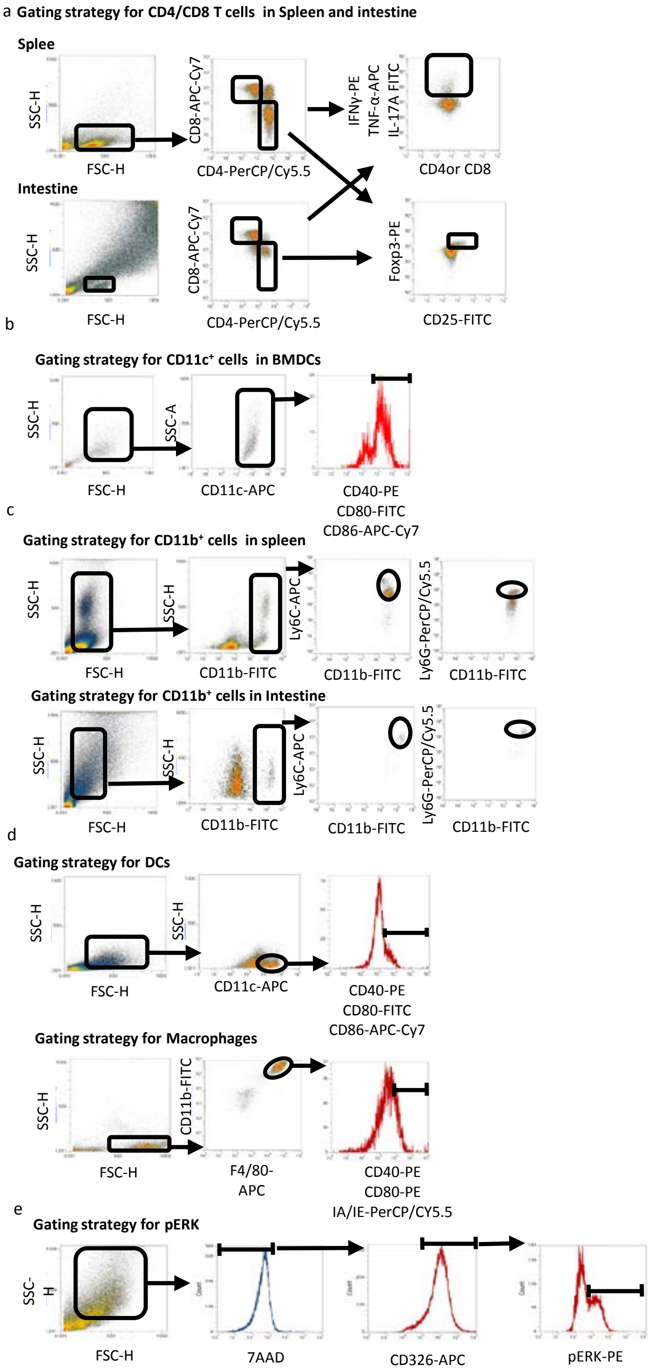

Supplementary Figure8: Gating strategies from Figure4A and 4B (a), Figure4D (b), Supplementary Figure3D (c), Supplementary Figure3G (d) and Supplementary Figure 4B (e).
